# Spatial–Temporal Heatmap Masked Autoencoder for Skeleton-Based Action Recognition

**DOI:** 10.3390/s25103146

**Published:** 2025-05-16

**Authors:** Cunling Bian, Yang Yang, Tao Wang, Weigang Lu

**Affiliations:** 1 Department of Education, Ocean University of China, Qingdao 266100, China; clbian@ouc.edu.cn (C.B.); yangyang2878@stu.ouc.edu.cn (Y.Y.); 2 Department of Campus Security, Ocean University of China, Qingdao 266100, China; wt@ouc.edu.cn

**Keywords:** skeleton-based action recognition, self-supervised learning, masked autoencoder, spatial–temporal heatmap, visual transformer

## Abstract

Skeleton representation learning offers substantial advantages for action recognition by encoding intricate motion details and spatial–temporal dependencies among joints. However, fully supervised approaches necessitate large amounts of annotated data, which are often labor-intensive and costly to acquire. In this work, we propose the Spatial–Temporal Heatmap Masked Autoencoder (STH-MAE), a novel self-supervised framework tailored for skeleton-based action recognition. Unlike coordinate-based methods, STH-MAE adopts heatmap volumes as its primary representation, mitigating noise inherent in pose estimation while capitalizing on advances in Vision Transformers. The framework constructs a spatial–temporal heatmap (STH) by aggregating 2D joint heatmaps across both spatial and temporal axes. This STH is partitioned into non-overlapping patches to facilitate local feature learning, with a masking strategy applied to randomly conceal portions of the input. During pre-training, a Vision Transformer-based autoencoder equipped with a lightweight prediction head reconstructs the masked regions, fostering the extraction of robust and transferable skeletal representations. Comprehensive experiments on the NTU RGB+D 60 and NTU RGB+D 120 benchmarks demonstrate the superiority of STH-MAE, achieving state-of-the-art performance under multiple evaluation protocols.

## 1. Introduction

Human action recognition poses a critical challenge in computer vision, involving the interpretation of human behaviors and discrimination between distinct actions. Prior research has explored diverse modalities for feature extraction, including RGB images [1,2], optical flow [3,4], audio signals [5], and skeletal data [6,7,8]. Advances in deep learning and pose estimation have significantly elevated the prominence of skeleton-based approaches. Skeletal data, typically represented as sequences of joint coordinates, encode pose information while remaining invariant to background clutter and lighting variations [9]. In skeleton-based action recognition, predominant methods rely on fully supervised learning frameworks employing Convolutional Neural Networks (CNNs) [9,10], Recurrent Neural Networks (RNNs) [11,12], Graph Convolutional Networks (GCNs) [13,14], and transformers [7,15]. Despite achieving notable performance, these methods are prone to overfitting and require labor-intensive annotated datasets.

To mitigate these challenges, self-supervised learning methodologies have gained traction in the field of skeleton action recognition [16,17]. These approaches utilize unlabeled data to derive meaningful representations. Some self-supervised strategies are centered on pretext tasks, such as reconstructing motions or solving jigsaw puzzles, to learn local features. However, these tasks may fall short in capturing comprehensive temporal dynamics. Conversely, contrastive learning techniques have emerged, training models to differentiate between positive and negative skeleton sequence pairs from varying perspectives [18,19,20]. These approaches prioritize high-level contextual understanding but may overly rely on the quantity of contrastive pairs, potentially overlooking inter-frame joint correlations.

A recent innovation in self-supervised learning is the introduction of masked autoencoders, which have demonstrated exceptional generalization capabilities and robust performance across diverse computer vision tasks [21,22]. This method involves obscuring substantial portions of the input image and training the model to reconstruct the original image using the visible portions. The original MAE approach employs an asymmetric encoder–decoder architecture, masking 75% of the input image to create a nontrivial self-supervisory task. However, while MAE excels in image-based tasks, applying this technique to human skeleton sequences presents distinct challenges due to the rich semantic content and complex temporal nature of these sequences.

To address the existing challenges in skeleton-based action recognition, we introduce an innovative self-supervised masked autoencoder framework known as the Spatial–Temporal Heatmap Masked Autoencoder (STH-MAE). Our STH-MAE framework extends the core MAE principles to spatio-temporal data by introducing a hierarchical masking strategy. Unlike MAE, which focuses solely on random patch masking, STH-MAE incorporates both spatial and temporal masking, capturing the intricate dynamics of skeleton sequences. Additionally, STH-MAE introduces a lightweight temporal decoder module that leverages the sequential nature of skeleton data, enabling more accurate reconstruction of both spatial relationships and temporal transitions. Effective masking strategies are thus critical to adequately capture the temporal dynamics and spatial relationships inherent in skeleton data, distinguishing STH-MAE’s approach from the original MAE framework.

The core structure of the STH-MAE pipeline is built on several fundamental principles. Initially, a spatial–temporal heatmap is created by integrating heatmaps across both spatial and temporal dimensions. During the pre-training phase, a unique spatial–temporal masking approach is employed, which segments the heatmap into uniform, non-overlapping patches and masks selected portions. To optimize spatial–temporal representation learning, we investigate various factors such as the masking ratio, heatmap resolution, training epochs, and the depth of the decoder to find an ideal balance between computational cost and performance. The encoder is designed to extract generalized feature representations from the input, while the decoder is responsible for reconstructing the masked heatmap patches. By exploiting the spatial–temporal properties of the heatmap, the autoencoder’s backbone is constructed based on the Vision Transformer (ViT) architecture, incorporating standard transformer blocks within an asymmetric encoder–decoder configuration. In the fine-tuning phase, only the encoder is employed, complemented by a simple output layer, to predict actions. Our method demonstrates superior performance compared to existing self-supervised learning techniques in skeleton-based action recognition, achieving competitive results without the need for additional data. This approach democratizes access to advanced action recognition capabilities, especially in scenarios where labeled data are scarce or expensive to obtain. Our key contributions can be summarized as follows:

- We introduce a streamlined and effective method for masked autoencoder in skeleton-based action recognition, emphasizing the acquisition of comprehensive and adaptable representations through the masking and reconstruction of skeleton sequences.
- We present a spatial–temporal heatmap as the primary representation of skeletons, enhancing robustness in pose estimation and capitalizing on recent advancements in Visual Transformers.
- Our model underwent extensive evaluation on the NTU RGB+D 60 and NTU RGB+D 120 datasets. Experimental results indicate that STH-MAE achieves state-of-the-art performance in self-supervised settings.

## 2. Related Works

### 2.1. Self-Supervised Skeleton-Based Action Recognition

Self-supervised skeleton-based action recognition has garnered considerable attention in research, with the primary objective of learning action feature representations from unlabeled skeleton sequences [23]. The advancements in this field can be broadly categorized into two approaches: those based on pretext tasks [16,24,25,26] and those utilizing contrastive learning techniques [27,28,29,30]. For instance, Zheng et al. [16] introduced a skeleton inpainting framework designed to capture long-term motion dynamics. Similarly, Lin et al. [24] employed multiple tasks, such as solving jigsaw puzzles, to learn more generalized skeleton features. Xu et al. [25] proposed a method involving reverse sequential predictions within an encoder–decoder structure to extract motion patterns. However, the feature representations derived from these methods might be overly specialized to the specific pre-designed tasks. In recent years, the incorporation of contrastive learning has led to significant progress in self-supervised skeleton-based action recognition. Contrastive learning has been effective in enhancing the learning of action representations by employing carefully crafted augmentations to generate varied movement patterns with consistent semantics. The primary challenge lies in applying robust augmentations that do not distort the skeletal structure to the point of semantic loss, which can result in unstable training. To mitigate this issue, Zhang et al. [29] introduced the Hierarchical Consistent Contrastive Learning framework, which integrates strong augmentations with a directional clustering mechanism to ensure hierarchical consistency in the learned representations. This framework gradually increases the complexity of augmentations and employs an asymmetric loss function. Additionally, Chen et al. [31] explored the significance of data augmentation and contrastive pair generation within the context of self-supervised skeleton-based action recognition. They developed SkeleMixCLR, a framework that utilizes spatio-temporal skeleton mixing augmentations to create challenging contrastive samples, thereby improving representation learning. This approach involves mixing two skeleton sequences and extending contrastive pairs with trimmed and truncated views. Research directions also focus on creating more robust and efficient methods for recognizing actions from skeleton sequences. Paoletti et al. [32] and Hua et al. [30] have highlighted the potential of unsupervised and part-aware contrastive learning in this domain. These methods emphasize high-level contextual information but often rely heavily on the quantity of contrastive pairs, potentially overlooking the correlation information between joints across frames. Leveraging advanced machine learning techniques, such as transformers, could further improve the ability of models to capture complex spatial and temporal relationships within skeleton sequences. In summary, learning task-specific representations that may not generalize to downstream action recognition, while contrastive learning based approaches require careful design of augmentations to avoid semantic distortion. This paper uses masked autoencoding, a task-agnostic framework, where reconstructing masked heatmap patches forces the model to learn comprehensive spatial–temporal dependencies.

### 2.2. Masked Autoencoder

In the realm of self-supervised learning, the masked autoencoder has emerged as a significant tool, allowing models to acquire meaningful data representations by inferring masked or omitted portions of the input. By leveraging the information from the visible parts of the input, this method enhances the model’s comprehension of the inherent data structure. Germain et al. [33] pioneered the Masked Autoencoder for Distribution Estimation, a technique that enables autoencoder outputs to be interpreted as conditional probabilities, significantly improving the generation of models with complete joint probability distributions. Zhou et al. [34] investigated the application of masked autoencoders for pre-training Vision Transformers in the context of medical image analysis, showing their efficacy in pre-training tasks involving natural image analysis. Geng et al. [35] introduced the Multimodal Masked Autoencoder, which facilitates the learning of transferable representations across visual and linguistic data without the need for modality-specific encoders. Zhang et al. [36] provided a comprehensive survey that underscored the increasing interest in masked autoencoders for self-supervised learning in vision and other domains, highlighting its potential as a promising avenue for self-supervised learning research. Further expanding on these concepts, Radosavovic et al. [37] explored the use of masked autoencoders in practical robot learning scenarios, while Yang et al. [38] examined their utility in domain-invariant self-supervised learning. Liu et al. [39] proposed Masked Decision Prediction as a self-supervised pre-training technique for reinforcement learning and behavioral cloning, utilizing masked autoencoders to reconstruct absent data in state–action trajectories. Reed et al. [40] introduced Scale-MAE, a scale-aware masked autoencoder designed for multiscale geospatial representation learning, emphasizing the importance of understanding relationships between data at various scales. Additionally, Woo et al. [41] and Wang et al. [42] extended the applicability of masked autoencoders to Convolutional Neural Networks and video pre-training, respectively, demonstrating the scalability and efficiency of masked autoencoders in developing foundational models for diverse data types. Overall, masked autoencoders have demonstrated versatility and effectiveness as tools for self-supervised learning and pre-training across various domains. Methods such as SkeletonMAE [43] apply masking to coordinate sequences, struggle with pose estimation noise and lack explicit spatial–temporal structure. Therefore, this paper introduces spatial–temporal heatmaps as the input representation, masking entire joints over time intervals, preserving structural relationships in spatial–temporal dimension.

### 2.3. Pose Estimation Heatmap

Human pose estimation represents a pivotal task within the domain of computer vision, aiming to accurately detect and pinpoint a person’s key points to comprehend posture and movement. In recent years, heatmap-based methodologies, which encode keypoint locations as heatmaps, have emerged as the leading approach in this field [44] and have proven particularly effective. These methods generate a probability distribution over the image, indicating the likelihood of each pixel being the keypoint. The key advantages of heatmap-based approaches include their ability to handle multiple keypoints simultaneously and their robustness to small perturbations in the image [45,46]. Despite the widespread adoption of heatmap-based regression in keypoint estimation tasks, including human pose estimation, this method exhibits significant limitations [47]. Comparative analyses between heatmap-based detection and integral regression for 2D pose estimation have shed light on crucial distinctions between these techniques [48]. Although heatmap regression predicts the likelihood of keypoint presence within a 2D space, it is critical to address the biases and inherent limitations of this approach [49]. Recent advancements in human pose estimation have also investigated sophisticated heatmap estimation structures, such as the MH Pose method, to improve both accuracy and performance [50].

While spatial–temporal data are widely applied across fields such as environmental monitoring [51], industrial systems [52], and causal discovery [52], our research targets skeleton-based action recognition, a domain marked by unique challenges including pose estimation noise and limited labeled data. Converting spatial–temporal data into heatmaps presents an engaging strategy, particularly for action recognition, as it simplifies complex data while preserving interpretability and minimizing information loss. Unlike Hu et al., who used spatial–temporal heatmaps in autonomous driving planning [53], our approach leverages heatmaps to encode joint probability distributions across spatial–temporal dimensions, thereby explicitly modeling human motion dynamics. Whereas Hu et al. applied heatmaps within a supervised learning framework for motion planning, our method, STH-MAE, employs a self-supervised masked autoencoder approach. By reconstructing masked heatmap patches, STH-MAE learns generalized representations without relying on labeled data, addressing the scarcity of annotated skeleton action sequences.

## 3. Proposed Method

**Figure 1:**
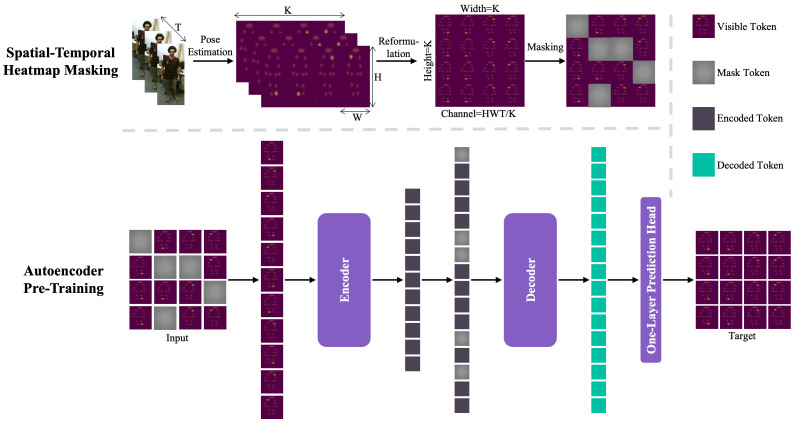
The proposed STH-MAE scheme for self-supervised skeleton-based action classification is depicted in the overall pipeline. At the top, the STH masking process is illustrated. Two-dimensional heatmaps are extracted from video frames and reformulated into an STH, which is then divided into patches that are randomly masked. The result is a set of K×K patches, each containing a 1D vector of length THW/K. The THW/K calculation is derived from flattening the spatial–temporal (T×H×W) dimensions. At the bottom, the autoencoder pre-training is shown. The encoder processes only the visible tokens, while mask tokens are added to the input sequence of the decoder to reconstruct the masked patches.

We aim to establish an efficient and streamlined framework tailored for self-supervised skeleton-based action classification representation learning via masked autoencoders. The proposed method, referred to as STH-MAE, is visually summarized in Figure 1. Initially, the input action sequence is processed through a Spatial–Temporal Heatmap Masking module. Following this, a Visual Transformer-based autoencoder, equipped with a single-layer prediction head, is utilized to reconstruct the masked segments of the spatial–temporal heatmap. Detailed explanations of STH-MAE are provided in subsequent sections.

### 3.1. Spatial–Temporal Heatmap Masking

**Figure 2:**
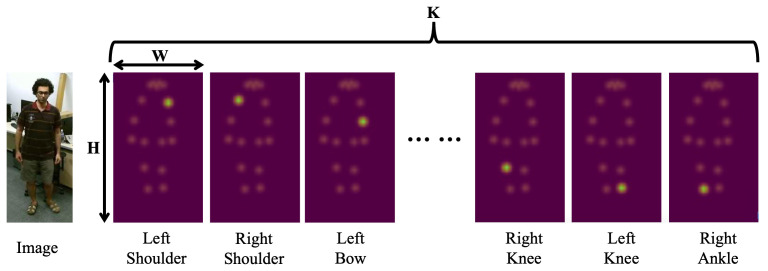
Heatmap structure for a single frame.

**Spatial–Temporal Heatmap.** The 2D poses extracted from the video frames are transformed for integration into STH-MAE by reshaping them into a spatial–temporal heatmap volume. Formally, a 2D pose is represented as a heatmap of dimensions K×H×W, where *T* denotes the temporal dimension representing the action sequence length, *K* corresponds to the anatomical joints in the human skeletal representation, and H×W specifies the spatial resolution of joint-specific confidence maps, as shown in Figure 2. Each heatmap encodes probabilistic joint localization through spatial coordinate likelihood estimation. The construction process involves two systematic phases. First, per-frame heatmap aggregation generates *K* distinct confidence maps, each of dimension H×W for every temporal instance t∈T, forming a 3D tensor K×H×W through channel-wise concatenation. Second, temporal integration stacks these frame-level tensors along the temporal axis via tensor concatenation, yielding the complete 4D STH representation T×K×H×W.

To ensure a consistent number of input patches for the encoder–decoder, the spatial–temporal heatmap is ultimately resized to THW/K×K×K, conforming it to an image-like format. The following steps are performed to reshape STH: Flatten Spatial-Temporal Dimensions combines *T*, *H*, and *W* into a single dimension; Partition into Tokens divides the flattened dimension by *K* to create THW/K dimensional vectors; Reshape into 3D Tensor then rearrange the flattened sequence into a 3D tensor with dimensions

(1) $$\mathrm{ST}H_{\mathrm{reshaped}\;}\in R^{K\times K\times \frac{THW}{K}}$$

where K×K is grid of tokens, equivalent to ViT’s patch grid and THW/K is the feature vector per token. The process transforms a 4D volume of sequential 2D poses into a 3D spatial–temporal heatmap volume, enabling the capture of both spatial and temporal dynamics of human actions. This representation is crucial for understanding complex motion patterns and interactions between different joints over time. Additionally, the heatmap volume can be easily integrated into transformer-based architectures, which have shown superior performance in capturing long-range dependencies in both spatial and temporal dimensions.

**Masking.** Following the method outlined in ViT [54], the spatial–temporal heatmap is divided into regular, non-overlapping K×K patches based on the joints and time intervals of the original 2D heatmap. Each patch comprises normalized 2D heatmaps of the same joint over a fixed time interval, which aids in convergence. A random sampling strategy is then employed to select a subset of these patches for masking, ensuring that patches are selected without replacement and follow a uniform distribution. Through experimentation, we have found that maintaining a relatively high, yet not extreme, masking ratio is most effective for our method. The set of masked tokens, denoted as $P_{gt}$, with a masking ratio *m*, is used as the ground truth for calculating the reconstruction loss. Our masking strategy draws inspiration from Vision Transformers but is tailored to the specific needs of skeleton-based action recognition. By partitioning the spatial–temporal heatmap into joint-time patches and employing a high masking ratio, we encourage the model to focus on learning comprehensive representations from limited visible information. This not only improves the model’s robustness to missing data but also enhances its ability to infer complex action patterns from partial observations.

### 3.2. Autoencoder Pre-Training

In our framework, the autoencoder is designed with a Vision Transformer (ViT) backbone, incorporating traditional transformer blocks and an asymmetric encoder–decoder architecture. The autoencoder’s final layer features a single-layer prediction head that aims to reconstruct the input.

**Encoder–Decoder.** Our approach applies the encoder only to unmasked patches, processing solely the visible tokens $T_{v}$ and excluding the mask tokens $T_{m}$. By focusing on a subset of the total tokens, we enable the training of considerably larger encoders with reduced computational and memory requirements. The unmasked tokens undergo linear projection embedding, with positional embeddings added, and are then processed through a sequence of transformer blocks, resulting in encoded tokens represented as $T_{e}$.

The decoder in our architecture mirrors the encoder but with a fewer number of transformer blocks. It accepts both encoded visible tokens $T_{e}$ and mask tokens $T_{m}$ as inputs. Each mask token, representing a missing patch to be predicted, is denoted by a shared, learned vector. Positional embeddings are incorporated for all tokens, including mask tokens, to maintain spatial location information. The integration of positional embeddings ensures that spatial information is preserved throughout the encoding and decoding processes, which is crucial for accurate reconstruction. The decoder processes these inputs to produce decoded mask tokens $H_{m}$, which are then forwarded to the prediction head. The encoder–decoder process can be summarized as follows:

(2) $$\begin{array}{cc} \; & T_{e}=\mathrm{Encoder}\left(T_{v}\right),\\ \; & H_{m}=\mathrm{Decoder}\left(\mathrm{concat}\left(T_{e},T_{m}\right)\right). \end{array}$$

During pre-training, the decoder is exclusively tasked with the reconstruction of masked tokens, while the encoder generates skeleton representations for action recognition. This separation allows for a flexible design of the decoder, independent of the encoder’s configuration. Our experiments explore various decoder sizes. The significance of our autoencoder’s architecture lies in its capacity to handle the complexity of visual data while optimizing computational efficiency. By exclusively focusing on visible tokens during encoding, we can allocate resources more effectively, enabling the training of larger models. Additionally, the flexibility in decoder design allows us to experiment with various configurations to determine the optimal balance between accuracy and computational overhead.

**Prediction Head.** Our approach, named STH-MAE, reconstructs the input image by predicting the pixel values for each masked patch. As the concluding layer of the autoencoder’s backbone, the prediction head’s purpose is to reconstruct the masked tokens. We utilize a fully connected layer as the prediction head. The output $H_{m}$ from the decoder is projected by the prediction head to a vector with dimensions matching the total number of pixels in a patch, followed by a reshape operation to generate the predicted masked tokens $P_{pre}$.

(3) $$\begin{array}{c} P_{pre}=\mathrm{Reshape}\left(\mathrm{FC}\left(H_{m}\right)\right). \end{array}$$

The fully connected layer within the prediction head is meticulously designed to handle high-dimensional data effectively, ensuring precise projection and reshaping operations.

**Reconstruction Target.** To compute the reconstruction loss, we compare the predicted masked tokens $P_{pre}$ with the actual masked tokens $P_{gt}$ using Mean Squared Error (MSE):

(4) $$\begin{array}{c} L=\frac{1}{N}\sum_{i=1}^{N}\left|\right|P_{pre}^{i}-P_{gt}^{i}{\left|\right|}^{2}, \end{array}$$

where *N* represents the number of pixels in a patch, and $P_{pre}^{i}$ and $P_{gt}^{i}$ are the predicted and ground-truth values for the *i*th pixel, respectively. The use of MSE as the loss function is crucial in minimizing the difference between the predicted and actual masked tokens, thereby optimizing the model’s performance.

## 4. Experiments

### 4.1. Datasets

The NTU-RGB+D 60 and NTU-RGB+D 120 datasets are the most prevalent datasets utilized in contemporary research.

**NTU RGB+D 60 (NTU 60)** is an extensive dataset designed specifically for the recognition of human actions based on skeletal data. It consists of 56,578 video sequences covering 60 different action categories, with each human figure represented by 25 joints. This dataset includes two standard benchmark protocols: Cross-Subject (X-Sub) and Cross-View (X-View).
**NTU RGB+D 120 (NTU 120)**, an extension of NTU 60, provides a larger dataset with 113,945 sequences encompassing 120 action labels. It also incorporates two benchmark protocols: Cross-Subject (X-Sub) and Cross-Set (X-Set). For these datasets, the estimation of 2D heatmaps for human joints is performed using HRNet [55] on sequences of RGB frames.

### 4.2. Evaluation Protocol

In our study, we employed the widely adopted evaluation protocols for these datasets.

**Linear Evaluation Protocol:** The models were evaluated using a linear evaluation approach for the action recognition task. This involves training a linear classifier (comprising a fully-connected layer followed by a softmax layer) with the encoder weights fixed.
**Semi-supervised Evaluation Protocol:** The encoder is pre-trained using the entire dataset and then the whole model is finetuned with only 1% or 10% randomly selected labeled data.
**Finetune Evaluation Protocol:** In this protocol, a linear classifier is appended to the pre-trained encoder, and the entire model is fine-tuned to compare its performance with fully supervised methods.

### 4.3. Implementation Details

All experiments were conducted using the PyTorch 1.9.0 deep learning framework on two RTX 3090 GPUs. Training employed the AdamW optimizer with a base learning rate of 0.001 and a weight decay of 0.05. The default mask ratio was set to 0.75, and the resolution was set to 8×8. The autoencoder’s backbone consisted of 24 transformer blocks for the encoder and 8 transformer blocks for the decoder. For the NTU 60 and NTU 120 datasets, all samples were reshaped to 85 frames. Additionally, sine–cosine positional embedding was added to both encoder and decoder inputs. During self-supervised representation learning, the autoencoder model was trained on the training set without ground-truth labels for 20 epochs. For supervised learning, the encoder model and linear classifier were randomly initialized and jointly trained on the training set with ground-truth action labels.

### 4.4. Ablation Study

This section delves into the analysis of key components and hyperparameters of STH-MAE. By systematically varying the masking ratio, heatmap resolution, decoder depth, and pre-training epochs, we can better understand the trade-offs and performance impacts of each parameter. Unless specified otherwise, all experiments are carried out on the NTU 60 dataset utilizing the Cross-Subject benchmarks and a linear evaluation protocol.

**Ablation study on masking ratio.** We investigated the effects of varying masking ratios on STH-MAE’s performance. Employing a random strategy, we tested patch masking ratios of 0.55, 0.75, and 0.95. As shown in Table 1, a masking ratio of 0.75 provides the best results among the tested ratios. Higher ratios foster exploration beneficial for tasks requiring extensive comprehension, whereas lower ratios emphasize exploitation, advantageous for detailed tasks. Achieving a balanced trade-off in the masking ratio enhances overall performance effectively.

**Ablation study on heatmap resolution.** The influence of 2D heatmap resolution on STH-MAE is examined, as illustrated in Table 2. Results demonstrate that the highest accuracy is obtained at a resolution of 8×8, consistent with expectations. Resolutions that are too low fail to capture features from ambiguous joints adequately, while excessively high resolutions struggle to discern complex spatial–temporal joint relationships. This balance is particularly critical for tasks involving complex spatial–temporal relationships, where both joint ambiguity and joint relationship complexity need to be effectively managed.

**Ablation study on decoder depth.** The decoder depth in our model refers to the number of transformer blocks employed. We tested different decoder depths, specifically 12, 8, and 4 blocks. Our experiments (refer to Table 3) reveal that STH-MAE achieves the highest performance with a Top1 accuracy of 84.31% when the decoder depth is set to 8 blocks. In contrast, deeper (12 blocks) and shallower (4 blocks) configurations result in lower performance, with Top1 accuracies of 83.47% and 82.90%, respectively. This difference can be attributed to the nature of the tasks involved—heatmap patch reconstruction versus recognition. In an autoencoder, the final layers focus primarily on reconstruction and are less critical for recognition. A deeper decoder captures these reconstruction nuances better, leading to more abstract latent representations. Understanding this balance can inform the design of more effective models for various tasks in computer vision. Moreover, as our STH-MAE framework employs a hierarchical transformer architecture, the computational complexity scales linearly with the number of input patches and transformer layers. However, our pre-training masking strategy, 75% patch masking, reduces the encoder’s effective input to 0.25 patches, lowering pre-training complexity by 4× compared to full-input training.

**Ablation study on pre-training epoch.** In the realm of self-supervised learning, it is commonly observed that extending the duration of pre-training epochs tends to yield more favorable outcomes. In our investigation, we systematically increased the pre-training epochs from 1 to 20, assessing optimal linear outcomes every 5 epochs. The data presented in Table 4 highlight that the highest accuracy, reaching 84.31%, was achieved at the 20-epoch mark, establishing it as the default setting. Noteworthy is the substantial enhancement of 4.3% observed between epochs 5 and 10. However, the subsequent progression from epochs 15 to 20 saw a marginal 0.1% improvement, signaling diminishing returns in efficacy beyond the 20-epoch threshold.

### 4.5. Comparison with State-of-the-Art

**Linear evaluation.** When comparing with current methods, the performance of STH-MAE stands out prominently, as shown in Table 5. Remarkably, STH-MAE consistently outperforms other approaches across all datasets evaluated. On the NTU 60 CS and NTU 120 X-sub datasets, it surpasses the previous leading method, ActCLR, by 3.4% and 5.3% respectively. This significant improvement highlights the efficacy of our spatial–temporal prediction targets.

**Fine-tuned evaluation.** The fine-tuning results, depicted in Table 6, highlight the efficacy of STH-MAE when trained with full supervision. On both the NTU 60 and NTU 120 datasets, STH-MAE not only matches but often exceeds the performance of the current state-of-the-art methods. Notably, while HYSP [60] holds strong performance metrics, STH-MAE enhances performance across almost all metrics, except for the X-sub on NTU 120.

**Semi-supervised evaluation.** Table 7 showcases the evaluation of semi-supervised results on the NTU 60 datasets. Particularly noteworthy is the performance of STH-MAE, which achieves a recognition rate of 39.8% for 1% of the data and 74.5% for 10% of the data on the CS benchmark. Similarly, on the CV benchmark, it achieves a recognition rate of 39.4% for 1% of the data and 77.2% for 10% of the data. It is important to highlight that STH-MAE not only matches but often surpasses the performance of the current state-of-the-art methods. Despite having limited labeled data, STH-MAE demonstrates significant performance improvements, showcasing its ability to learn effectively even in scenarios with less supervision. This capability of STH-MAE to perform well under such conditions is critical for real-world applications, especially where labeled data are scarce or expensive to acquire. Consequently, STH-MAE’s adeptness in delivering high performance under these circumstances makes it exceptionally valuable for practical implementations, ensuring that optimal performance can be maintained even with limited resources.

STH-MAE achieves state-of-the-art performance in most benchmarks, though minor variations arise due to inherent trade-offs in self-supervised learning. As a reconstruction-based framework, STH-MAE, prioritizes generalizable spatial–temporal dependencies, which excel in robustness and label efficiency but may lag slightly in highly specialized tasks requiring fine-grained distinctions. This reflects the balance between generality and specificity in representation learning, suggesting future exploration of adaptive strategies or hybrid objectives to address such gaps while retaining computational advantages.

## 5. Conclusions

In this study, we propose STH-MAE, a novel self-supervised framework for skeleton-based action recognition that leverages spatial–temporal heatmap reconstruction to learn comprehensive skeletal representations. Our approach begins by aggregating 2D heatmaps across spatial and temporal dimensions to construct a unified spatial–temporal heatmap. A randomized masking strategy is then applied to occlude patches within this heatmap, followed by reconstruction using a Visual Transformer architecture with a single-layer prediction head. Through this process, the model learns transferable skeletal features without requiring labeled data. Comprehensive evaluations on two large-scale benchmarks validate the effectiveness of STH-MAE, achieving state-of-the-art results across multiple metrics and outperforming existing methods.

Future research could extend this work by integrating multi-modal data to enhance action recognition robustness, or exploring cross-domain transfer learning strategies through large-scale pre-training followed by task-specific fine-tuning. These directions may further advance the adaptability and performance of skeleton-based models in real-world applications.

## Figures and Tables

**Table 1:** Ablation study on masking ratio.

Masking Ratio	0.55	0.75	0.95
Top1	82.84	**84.31**	78.65
Top5	96.08	**98.33**	95.24

Bold numbers indicate the best results in each task.

**Table 2:** Ablation study on heatmap resolution.

Resolution	4 × 4	8 × 8	16 × 16	32 × 32	48 × 48
Top1	82.68	**84.31**	81.98	78.42	71.03
Top5	97.97	**98.33**	97.85	96.97	94.60

Bold numbers indicate the best results in each task.

**Table 3:** Ablation study on decoder depth.

Depth	4	8	12
Top1	82.90	**84.31**	83.47
Top5	98.23	**98.33**	98.19

Bold numbers indicate the best results in each task.

**Table 4:** Ablation study on pre-training epoch.

Pre-Training Epoch	1	5	10	15	20
Top1	70.01	79.82	83.22	84.21	**84.31**
Top5	94.45	97.53	98.15	98.36	**98.33**

Bold numbers indicate the best results in each task.

**Table 5:** Linear evaluation results with state-of-the-art methods on NTU 60 and NTU 120 datasets.

Method	Year	NTU 60		NTU120	
		CS	CV	X-Sub	X-Set
LongT GAN [16]	AAAI18	39.1	48.1	-	-
MS2L [24]	ACM MM20	52.6	-	-	-
AS-CAL [56]	IS21	58.5	64.8	48.6	49.2
P&C [17]	CVPR20	50.7	76.3	42.7	41.7
SeBiReNet [57]	ECCV20	-	79.7	-	-
SkeletonCLR [27]	CVPR21	75.0	79.8	60.7	62.6
Colorization [26]	ICCV21	75.2	83.1	-	-
CrossSCLR [27]	CVPR21	77.8	83.4	67.9	67.1
AimCLR [28]	AAAI22	78.9	83.8	68.2	68.8
CMD [58]	ECCV22	79.8	86.9	70.3	71.5
PSTL [59]	AAAI23	79.1	83.8	69.2	70.3
HiCRL [29]	AAAI23	80.4	85.5	70.0	70.4
HaLP [20]	CVPR23	79.7	86.8	71.1	72.2
HYSP [60]	ICLR23	78.2	82.6	61.8	64.6
ActCLR [61]	CVPR23	80.9	86.7	69.0	70.5
skeleton-logoCLR [62]	TSCVT24	82.4	**87.2**	72.8	73.5
**STH-MAE (Ours)**	-	**84.3**	87.0	**74.3**	**75.6**

Bold numbers indicate the best results in each task.

**Table 6:** Fine-tuned evaluation results with state-of-the-art methods on NTU 60 and NTU 120 datasets.

Method	Year	NTU 60		NTU120	
		CS	CV	X-Sub	X-Set
SkeletonCLR [27]	CVPR21	82.2	88.9	73.6	75.3
AimCLR [28]	AAAI22	83.0	89.2	76.4	76.7
SkeletonMAE [43]	ICMEW23	86.6	92.9	76.8	79.1
HYSP [60]	ICLR23	86.5	93.5	**81.4**	82.0
ActCLR [61]	CVPR23	85.8	91.2	79.4	80.9
skeleton-logoCLR [62]	TSCVT24	86.1	93.6	79.2	80.0
**STH-MAE (Ours)**	-	**89.8**	**94.9**	80.1	**83.5**

Bold numbers indicate the best results in each task.

**Table 7:** Semi-supervised evaluation results with state-of-the-art methods on NTU 60 dataset.

Method	Year	1% Data		10% Data	
		CS	CV	CS	CV
LongT GAN [16]	AAAI 2018	35.2	-	62.0	-
ASSL [63]	ECCV 2020	-	-	64.3	69.8
MS2L [24]	ACM MM 2020	31.1	-	65.2	-
MCC [64]	ICCV 2021	-	-	60.8	65.8
Skeleton-Contrastive [65]	ACM MM 2021	35.7	38.1	65.9	72.5
Hi-TRS [66]	ECCV 2022	39.1	**42.9**	70.7	74.8
**STH-MAE (Ours)**	-	**39.8**	39.4	**74.5**	**77.2**

Bold numbers indicate the best results in each task.

## Data Availability

Restrictions apply to the availability of these data. Data were obtained from Rose Lab and are available https://rose1.ntu.edu.sg/dataset/actionRecognition/ (accessed on 2 April 2025) with the permission of Rose Lab.
